# Magnetic Field Is the Dominant Factor to Induce the Response of *Streptomyces avermitilis* in Altered Gravity Simulated by Diamagnetic Levitation

**DOI:** 10.1371/journal.pone.0024697

**Published:** 2011-10-19

**Authors:** Mei Liu, Hong Gao, Peng Shang, Xianlong Zhou, Elizabeth Ashforth, Ying Zhuo, Difei Chen, Biao Ren, Zhiheng Liu, Lixin Zhang

**Affiliations:** 1 Chinese Academy of Sciences Key Laboratory of Pathogenic Microbiology and Immunology, Institute of Microbiology, Chinese Academy of Sciences (CAS), Beijing, People's Republic of China; 2 Graduate University of Chinese Academy of Sciences, Beijing, People's Republic of China; 3 Key Laboratory for Space Biosciences & Biotechnology, Faculty of Life Sciences, Northwestern Polytechnical University, Xi'an, People's Republic of China; Massachusetts Institute of Technology, United States of America

## Abstract

**Background:**

Diamagnetic levitation is a technique that uses a strong, spatially varying magnetic field to simulate an altered gravity environment, as in space. In this study, using *Streptomyces avermitilis* as the test organism, we investigate whether changes in magnetic field and altered gravity induce changes in morphology and secondary metabolism. We find that a strong magnetic field (12T) inhibit the morphological development of *S. avermitilis* in solid culture, and increase the production of secondary metabolites.

**Methodology/Principal Findings:**

*S. avermitilis* on solid medium was levitated at 0 g*, 1 g* and 2 g* in an altered gravity environment simulated by diamagnetic levitation and under a strong magnetic field, denoted by the asterix. The morphology was obtained by electromicroscopy. The production of the secondary metabolite, avermectin, was determined by OD_245 nm_. The results showed that diamagnetic levitation could induce a physiological response in *S. avermitilis*. The difference between 1 g* and the control group grown without the strong magnetic field (1 g), showed that the magnetic field was a more dominant factor influencing changes in morphology and secondary metabolite production, than altered gravity.

**Conclusion/Significance:**

We have discovered that magnetic field, rather than altered gravity, is the dominant factor in altered gravity simulated by diamagnetic levitation, therefore care should to be taken in the interpretation of results when using diamagnetic levitation as a technique to simulate altered gravity. Hence, these results are significant, and timely to researchers considering the use of diamagnetic levitation to explore effects of weightlessness on living organisms and on physical phenomena.

## Introduction

Understanding how the space ‘condition’ influences bacterial behaviour is important, not only for the immediate health of astronauts, but also for the long-term future of space exploration. Spaceflight has been reported to affect microbial cellular processes, such as cell growth [1], gene expression [2], and so on, therefore potentially producing unexpected (and possibly dangerous) changes in the natural biota within the space craft. For earth based organisms, microgravity is a key factor of the space condition, since all earth based organisms have been evolving under a 1g field for millions of years. Thus, in recent years, and as space technology develops, studies concerning the influences of microgravity on microbial cells are drawing more and more attention, and knowledge regarding microbes' adaptations in the space environment are sort.

Because of the rarity and costliness of experiments in orbit, studies of the effect of the space environment on biology are limited to a large degree. To overcome issues with experiments in space, and in accordance with the hypothesis that “sensing no weight” would have comparable effects to those of weightlessness [3], several forms of ground-based simulated setups that simulate weightlessness have been developed. Many of these experiments have used rotation to time-average the gravity vector to zero, however this can introduce artifacts owing to the rotating reference frame (clinostats, random positioning machine) [4].

For the results in this paper we used diamagnetic force to simulate variable gravity environments [5]. Just as the centrifugal force balances the gravitational force on an orbiting spacecraft, the diamagnetic force opposes the force of gravity on a levitating object. In previous works, diamagnetic force has been applied to levitate protozoan [6], plants [7], and even mammals [8]. Effects of altered gravity environments simulated by diamagnetic levitation on protein crystallization [9], plasmid transfer [10], cell growth and gene expression [11] have been previously investigated. Apart from centrifugation experiments in space, it is the only available method that can simulate both hypergravity and hypogravity in a single experimental setup. This quality permits direct comparative studies of the artifacts of these conditions on individual samples. However, in common with all ground-based techniques to simulate weightlessness, there are effects introduced by diamagnetic levitation that are not present in a weightless environment. It has been demonstrated that the magnetic field that levitates the cell also induces convective stirring in liquid media, thus enhances the oxygen availability by transporting oxygen more effectively around a liquid culture [4]. Therefore the use of solid medium should be used to prevent the effects, and artifacts, of magnetically driven convection of oxygen.

In this study, we chose *Streptomyces avermitilis* as the test organism. *S. avermitilis* has been used for industrial production of the important anthelmintic agent avermectin, and has already been proven to be a highly efficient producer of secondary metabolites [12]. Of the eight major avermectin compounds, B1a is the most efficient component against a broad range of nematodes and arthropod parasites of domestic animals [13]. Here, for the first time, we assess whether magnetic field and variations in gravity have independent effects on morphology and secondary metabolism of *S. avermitilis* in solid culture, and thus determine whether diamagnetic levitation is a suitable technique to simulate the altered gravity environment.

## Materials and Methods

### Microorganism and media

Strain *S. avermitilis* PE1, was a high producing mutant isolated and identified after N-methyl-N0-nitroso-N-nitrosoguanidine (NTG) treatment in our laboratory, and grown on YMG agar medium. *S. avermitilis* was grown for fermentation, and HPLC assay was performed as previously described [14].

### Apparatus

**Figure 1:**
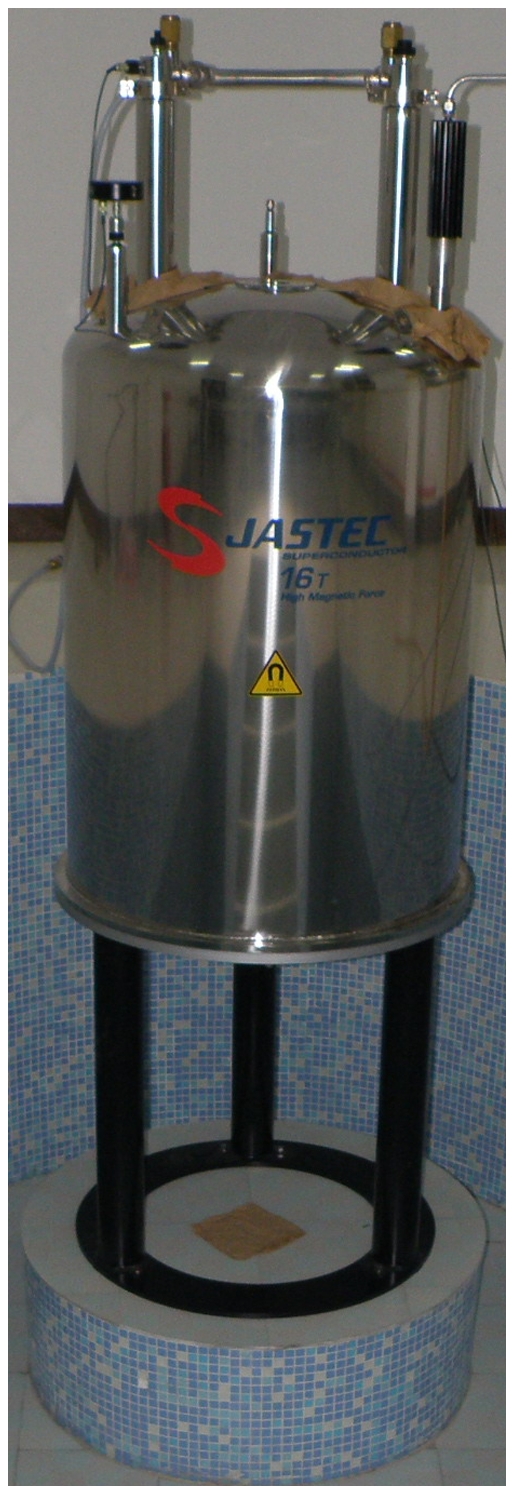
Photograph of the diamagnetic levitation apparatus. The apparatus is located in the Key Laboratory for Space Biosciences & Biotechnology of the Northwestern Polytechnical University, China.

The experiments were carried out using a ground-based bioreactor within a large gradient superconducting magnet, developed by the Northwestern Polytechnical University, China (Fig. 1). The magnet had a 51-mm vertical bore and produced a 16.12-T maximum field. The experimental samples were viewed with a subminiature CCD camera (QN42HL, EMLO, USA), and images were recorded with a computer. The temperature of the chambers containing the samples was kept at 28°C using a digital water-circulating bath, with variation over time of less than 0.5°C. A water jacket was installed in the bore to guarantee minor temperature fluctuation, and brass was chosen for the wall of the water jacket to allow the temperature control system to continue to work at a high magnet field. The temperature-controlled chamber consisted of a tube, inserted into the magnet bore, with three slots, one at each of the 0 g*, 1 g* and 2 g* gravitational positions (the asterisk on the label indicated the sample was in a strong magnetic field). The 1 g* sample was located at the centre of the coil, since at this point, the sample was not exposed to any change in normal gravitational force; the 0 g* sample was located above the center, where the magnetic force balanced the gravitational force; and the 2 g* sample was located below the 1 g* position, where gravity and the magnetic force were additive [15]. A control‘1 g’ sample was also included, which is grown outside of the magnet and compared to the 1 g* sample. This enabled us to distinguish experimentally between the effects of magnetic forces and that of altered gravity.

### Levitation protocol

The spores of *S. avermitilis* strain PE1 were suspended in sterile water, divided into four aliquots for the four test groups (1 g and three experimental groups: 0 g*, 1 g*, and 2 g*), and spread onto four identical YMG plates. These plates were then inserted into the corresponding slots in the bore and grown at 28°C for 7 days. The 1 g group was grown at 28°C outside the magnet.

### Identification of mutants

After levitation, the samples were serially diluted in sterile water and streaked on YMG plates until single colonies were isolated. Single colonies were selected randomly and inoculated into a 96-well plate containing solid medium, and cultured for 10 days. Analysis of the absorbance at 245 nm, the characteristic absorption wavelength of avermectin, was carried out on a plate reader. The positive mutants were identified as those with a >10% increase in the OD_245 nm_ value, when compared with the original strain. The negative mutants were identified as those with a >10% decrease in OD_245 nm_, when compared with the original strain. The positive/negative mutation ratio was calculated as the number of positive/negative mutants divided by the total number of screened colonies.

### Statistical analysis

Data are expressed as mean±standard error throughout the manuscript. Statistical analysis was performed using SPSS 11 (SPSS, Chicago, IL, USA). A value of *P*<0.05 was considered statistically significant in all cases.

## Results

### Effects of magnetic field and gravity on morphology and growth of *S. avermitilis*

**Figure 2:**
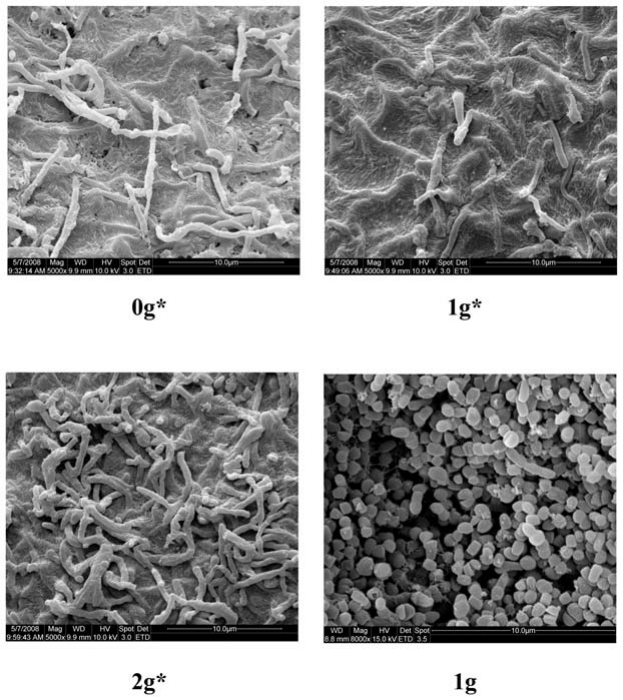
The effect of magnetic field on mycelium morphology of *S. avermitilis*. The electron microscopic photos showed retardation of the morphological shift from filamentous to coccid, after magnetic levitation.

Figure 2 shows the effect of magnetic field and gravitational force on the mycelium morphology of strain PE1. We found that after 7days the cells of the 1 g group were sporulating, while those of the 1 g* group, exposed to the 12T magnetic field, remained filamentous. The mycelium of 1 g was also more abundant than that of 1 g*. The mycelium was less dense for groups 0 g* and 1 g* group compared to that of 2 g*. As shown in Figure 2, the magnetic field had negative effect on the morphology of PE1, and the effect was more significant than that of gravitational force. Furthermore, increasing the gravitational force did not significantly affect the morphology of PE1, but could compensate the negative effect of magnetic field in part. These results demonstrated that we could isolate and quantitate a direct effect of the high magnetic field and of gravity on the morphology of *S. avermitilis* strain PE1.

### Effects of magnetic field and gravity on avermectin productivity

**Figure 3:**
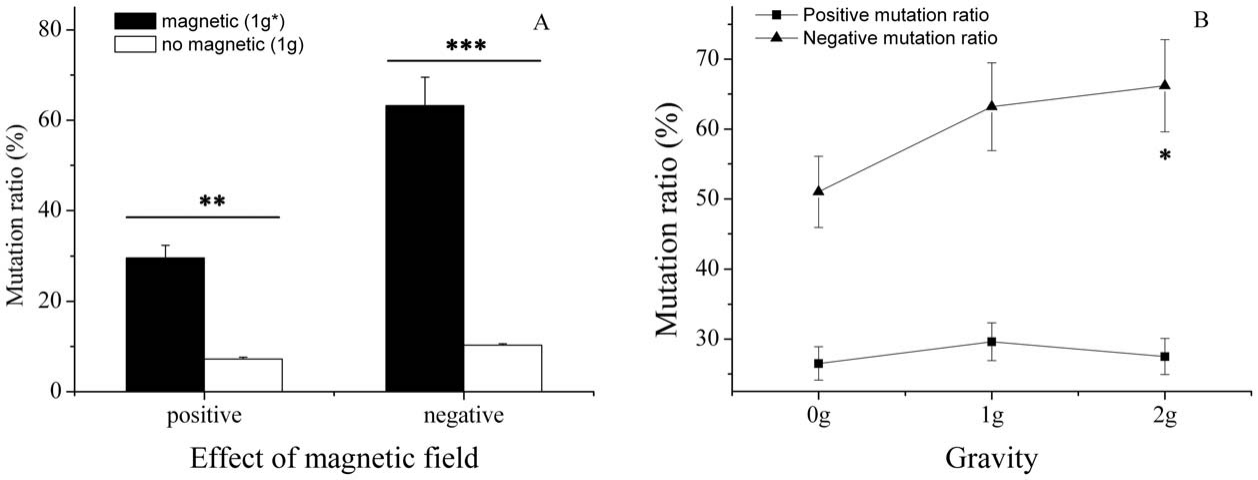
Effect of magnetic field (A) and gravity (B) on mutation ratios. ***P<0.001, **P<0.01, *P<0.05.

Significant changes in the avermectin production profile of PE1 were seen after levitation. The strong 12T magnetic field caused the positive mutation ratio to increase from 7.2±0.4% in group 1 g, to 29.6±2.7% in 1 g* (Fig. 3A). The negative mutation ratio increased from 10.1±0.3% in the group 1 g, to 63.2±6.3% in 1 g*, suggesting magnetic field promoted the instability inherent in *Streptomyces* [16]. An increase in gravitational force (Fig. 3B) was found to cause an increase in the percentage of negative mutants: 51.0±5.3% at 0 g*; 63.2±6.3% at 1 g*; and 66.1±6.3% at 2 g*. The positive mutation ratio remained more stable with an increase in gravitational force: 26.5±2.4% at 0 g*; 29.6±2.7% at 1 g*; and 27.5±2.6% at 2 g*. These results demonstrated that changes in avermectin production could be mainly attributed to the magnetic field rather than altered gravity environment, especially those mutations resulting in positive increases in avermectin production.

One mutant, PE11, obtained from the 0 g* group, produced a particularly high yield of avermectin. The production of avermectin of PE11 remained stable during 4 generations of flask culture (Table 1), and therefore could provide a stable technique for improving yield by mutation.

**Table 1:** The genetic stability of the mutant strain PE11 (avermectin yield of original strain was 100.0±6.77%).

Generation	Avermectin yield relative to original strain PE1, %
1	128.0±3.01
2	122.1±3.35
3	124.2±4.08
4	126.4±3.69

## Discussion

Several reports on changes in secondary metabolism during rotary wall vehicle suspension culture exist [17], however, they do not differentiate the effects of rotary force and microgravity. Valles and Guevorkian (2002) have described the use of magnetic field gradient levitation apparatus as a low gravity simulator for biological systems [18]. Diamagnetic levitation certainly has the potential to be a powerful tool to study the effects of weightlessness on biological samples, to complement existing ground-based techniques, however artifacts may exist.

In this study, we have investigated, for the first time, the individual effects of magnetic field and gravitational force on the morphology and secondary metabolism of *S. avermitilis*. From our experimental data, we discovered that the physiological response of strain PE1 to magnetic field exposure resulted in suppression of sporulation and a reduction in mycelium at 12T. There have been few reports on the effect of magnetic field on microbe growth. Zhang, et al. (2002) observed that the growth of *E. coli* was inhibited due to the presence of magnetic field of up to 0.6 T, the maximum used in that experiment [19]. Iwasakaa et al. (2004) observed that the rate of yeast proliferation decreased after 16 h of incubation under a high magnetic field (14T) compared to the control group [20]. Ji et al. (2010) also found that high-level magnetic field (>200 mT) inhibited the growth of mixed bacteria of activated sludge [21]. While these results confirm our own findings, mechanisms that explain how magnetic fields may initiate changes in biological systems have not yet been elucidated.

Other reports have focused on the mutagenic effects of static magnetic fields. Four strains of *Salmonella typhimurium* (TA98, TA100, TA1535 and TA1537) and *Escherichia coli* WP2 *uvrA* were exposed to a high magnetic field (5T) [22]. The *uvrA,* which lacked the UvrA protein that functions in the initial step of nucleotide excision repair but had normal activity to protect the cells against oxidative stress showed no mutagenic potential [24]. Zhang, et al (2003) examined the effect of strong static magnetic field (up to 10T) on various *E. coli* mutants defective in repair of oxidative DNA damage, redox regulation, defence systems against oxidative stress. Only the mutation in the mutants defective in defence mechanisms against oxidative stress was significantly enhanced in an exposure-dependent manner [23]. In this work, we detected the mutation effect of a high magnetic field on *S. avermitilis* strain PE1. *Streptomyces* lives in the soil and are often challenged with diverse environmental stresses [24] which always trigger cell morphological differentiation associated with secondary metabolism [25]. The strain PE1, used in this study, was obtained by serially random mutation in order to obtain a high titer of avermectin production. The mutation effect of the magnetic field on strain PE1 suggests that, the defense mechanism for oxidative stress or other stress factors in strain PE1 might have been disabled by these purposeful mutations prior to this experiment.

Results presented here suggest that the effects of gravity can be differentiated from the effects of magnetic field alone. This study showed that with diamagnetic levitation, magnetic field, rather than gravity, is the dominant factor inducing physiological response in strain PE1. The authors thus suggest caution to be used when analyzing results where diamagnetic levitation is used to simulate a microgravity environment.
